# The Clinical Spectrum of Hypophosphatasia in Older Adults

**DOI:** 10.1002/ccr3.70920

**Published:** 2025-10-30

**Authors:** Estefania Valdez Navarro, Evelyn M. Wong, Lianne Tile, Angela M. Cheung

**Affiliations:** ^1^ Osteoporosis Program University Health Network (UHN) Toronto Ontario Canada; ^2^ Department of Medicine University Health Network and Sinai Health System, University of Toronto Toronto Ontario Canada

**Keywords:** aging, endocrinology and metabolic disorders, genetics and genomics, geriatrics

## Abstract

Alkaline phosphatase (ALP) should be measured in older adults presenting with fragility fractures. Hypophosphatasia (HPP) should be suspected in individuals with hypophosphatasemia (low serum ALP). A correct diagnosis allows clinicians to avoid using potent antiresorptive osteoporosis medications, which are contraindicated in patients with HPP.

## 1 Introduction

Fragility fractures, defined as bone fractures that occur with minor or no trauma, are a significant cause of morbidity in the older adult population (age 65 and older). These result in excess mortality, lower quality of life, and significant costs to the health care system. The incidence of fragility fractures is increasing as life expectancy increases. While most fragility fractures in the older population are attributed to osteoporosis, it is crucial to rule out secondary causes in order to direct treatment.

HPP is a genetic disorder caused by a mutation in the ALPL gene, impairing the function of the tissue‐nonspecific isoenzyme of alkaline phosphatase (TNSALP) [1]. Due to the enzyme defect, the TNSALP substrate inorganic pyrophosphate (PPi) inhibits bone mineralization, causing osteomalacia [2]. HPP in older patients is an overlooked diagnosis that may be missed because low bone mass and fragility fractures are commonly labeled as osteoporosis, without further investigation. ALP should be measured as a component of the secondary workup, and if low, further investigation is needed.

As HPP causes osteomalacia, potent antiresorptive osteoporosis medications (bisphosphonates and denosumab) are contraindicated. Bisphosphonates are PPi analogs which further exacerbate mineralization defects in hypophosphatasia, where endogenous PPi is already elevated. Denosumab inhibits osteoclast activity by inhibiting RANK ligand. Both classes of antiresorptives decrease bone resorption, which in turn decreases TNSALP activity and levels [1, 3].

We present two cases of adult HPP in patients diagnosed in their 70s, highlighting the spectrum of clinical presentations in older adults. This can range from asymptomatic disease to fragility fractures. These cases emphasize the importance of an accurate diagnosis in order to guide treatment in those with less common causes of fractures.

## 2 Case Presentation: Patient 1

Our first case is a 72‐year‐old woman who was referred by her primary care provider because of a low ALP level (hypophosphatasemia). She had known low bone mass since her 50s. She had been on treatment with oral bisphosphonates for 14 years for osteoporosis until age 62, when she experienced a wrist fracture after a fall from standing height. Due to presumed treatment failure, she was switched to denosumab, which she took for 4 years. Denosumab was then stopped by the prescribing physician, without sequential therapy, after BMD gains were seen.

A hip replacement was performed in her 50s for osteoarthritis. She experienced menopause at the age of 40, with no hormone replacement therapy afterward. She had no early dental issues, no childhood arthralgias or fractures, and there is no family history of fractures. Physical examination was unremarkable.

### 2.1 Investigations and Treatment: Patient 1

She had a low ALP of 26 IU/L (reference range 44–147 IU/L), with low ALP on testing dating back at least a decade. Other test results are shown in Table 1. Bone turnover markers were not available. Bone mineral density (BMD) was lowest at the left femoral neck, with *T*‐score −2.4.

**TABLE 1:** Relevant biochemical findings.

Test	Case 1	Case 2	Reference range
ALP	26 IU/L	21 IU/L	44–147 IU/L
Vitamin B6	20.8 μg/L	228 nmol/L	2–21.7 μg/L 20–96 nmol/L
Calcium	2.49 mmol/L	2.60 mmol/L	2.2–2.62 mmol/L
Phosphate	1.37 mmol/L	1.08 mmol/L	0.80–1.40 mmol/L
25‐OH Vitamin D	97 IU/L	106 IU/L	25–200 nmol/L
PTH	3.8 pmol/L	3.4 pmol/L	2.0–9.4 pmol/L
Creatinine	58 μmol/L	92 μmol/L	64–110 μmol/L
TSH	1.67mlU/L	1.67 mlU/L	0.32–4.00 mIU/L

Although vitamin B6 levels were in the normal range, her persistently low ALP levels, history of fragility fracture, and low bone density, a presumptive diagnosis of HPP was made, and genetic testing was conducted. This showed a heterozygous pathogenic c.881A>C variant (p.Asp294Ala) in the ALPL gene, confirming the diagnosis.

As HPP causes osteomalacia, potent antiresorptive osteoporosis medications (bisphosphonates and denosumab) are contraindicated, because they can worsen osteomalacia.

Enzyme replacement therapy was discussed with the patient. She declined for reasons that included need for frequent injections and limited long‐term safety data. Furthermore, she was diagnosed in adulthood with no childhood signs of the condition, restricting access to coverage. Taking into account patient preference, our decision was for clinical surveillance, adequate vitamin D supplementation and calcium intake through her diet, as well as balance and resistance exercises. Anabolic osteoporosis therapy could be considered in the event of future fractures.

### 2.2 Outcome and Follow‐Up: Patient 1

During a three‐year follow‐up, no new fractures were reported, and she continued with her daily activities. The patient had no offspring, but she expressed concern about a brother diagnosed with osteoporosis and currently being treated with denosumab, prompting advice for genetic testing for him.

## 3 Case Presentation: Patient 2

The second case is a 76‐year‐old man in whom hypophosphatasemia was incidentally identified during routine bloodwork with a new primary care provider, prompting referral to our clinic. BMD was normal. He did not have a history of fragility fractures and he had never received osteoporosis medications. Medical history was significant for type 2 diabetes, hypertension and dyslipidemia. Physical examination was unremarkable.

### 3.1 Investigations and Treatment: Patient

ALP was 21 U/L (range 40–129 U/L); and review of records confirmed low ALP for over a decade. Other results included in Table 1. Bone turnover markers as follows: C‐telopeptide 131 (118–776 ng/L) and osteocalcin 8 (13‐48 μg/L). BMD was lowest at the left femoral neck, with *T*‐score −0.1. Genetic testing revealed a heterozygous likely pathogenic variant c.650delinsCTAA (p.Val217delinsAlaLys) in the ALPL gene.

### 3.2 Outcome and Follow‐Up: Patient 2

Similar to patient 1, we advised against using potent antiresorptive osteoporosis medications (bisphosphonates and denosumab) in case of low BMD or fragility fractures during follow‐up. He was managed with adequate vitamin D supplementation and dietary calcium intake, as well as balance and resistance exercises. Over 2 years of follow up he remained asymptomatic, and no fragility fractures were reported. Enzyme replacement therapy was discussed; however he declined due to lack of symptoms. He also expressed concern about a brother who was previously treated for osteoporosis with a bisphosphonate. This led to a discussion about disclosing this diagnosis to his relatives.

## 4 Discussion

HPP is a genetic disorder caused by a mutation in the ALPL gene, impairing the function of TNSALP [1]. Due to the enzyme defect, the TNSALP substrate inorganic pyrophosphate (PPi) inhibits bone mineralization, causing osteomalacia [2]. Clinical manifestations vary from severe neonatal disease to a mild presentation in the adult population, often presenting in mid‐life with multiple fractures [4, 5, 6].

**FIGURE 1:**
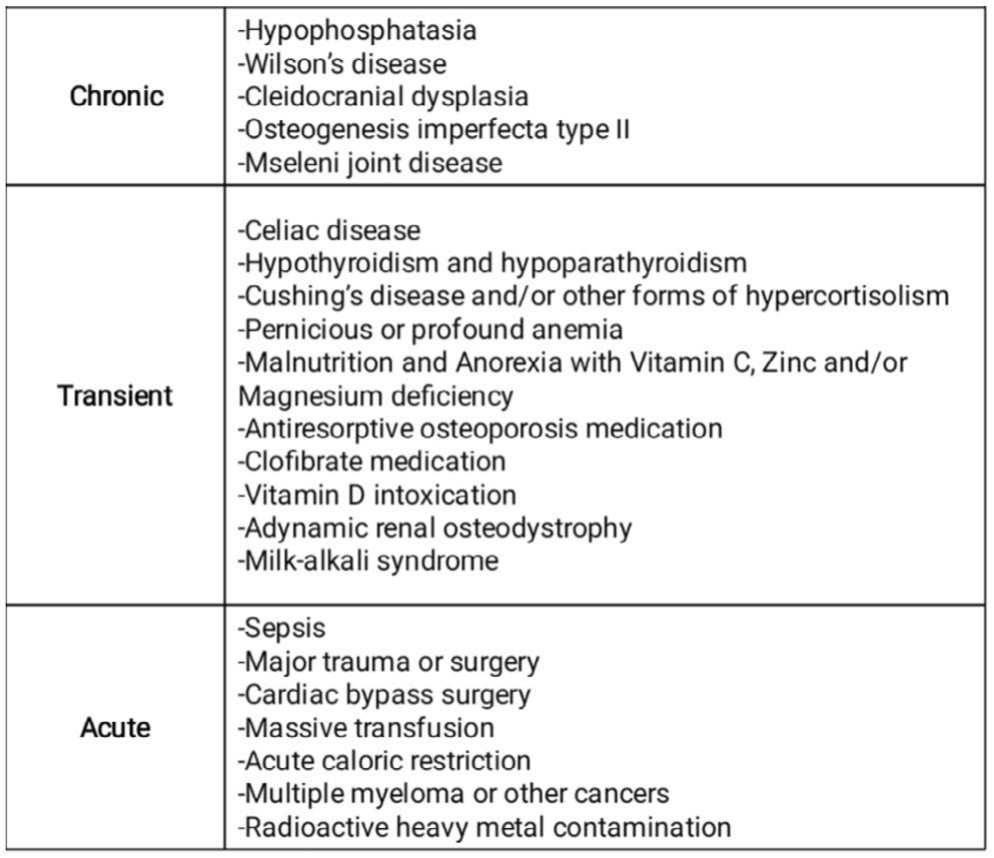
Causes of low ALP. Author's original work.

Prevalence in the adult population ranges from 1:275 to 1:6370, with a median age at diagnosis of 49 years [7, 8]. Hypophosphatasia in older patients is a diagnosis that may be missed, because low bone mass and fragility fractures are commonly labeled as osteoporosis. ALP should be measured as a component of the secondary workup, and if hypophosphatasemia is present, further investigation is needed. Other causes of low ALP levels can be found in Figure 1 [9].

Hypophosphatasemia may be observed in patients receiving antiresorptive therapies. While mild decreases in ALP may be related to treatment, persistently low levels, particularly in the context of musculoskeletal symptoms or atypical fractures, should prompt consideration of hypophosphatasia. The degree and chronicity of ALP suppression may help distinguish between benign treatment‐related changes and underlying HPP. Both cases presented here had a long history of hypophosphatasemia, which prompted HPP genetic investigations.

While genetic testing can confirm the diagnosis when pathogenic ALPL mutations are found, a negative result does not exclude HPP, as there is a growing database of pathogenic variants. In such cases, diagnosis remains clinical, supported by biochemical markers such as low ALP and elevated vitamin B6 levels. An important aspect to consider in elderly patients is that while an elevated vitamin B6 is a helpful biochemical marker in HPP, it may be confounded by exogenous intake. A careful review of supplements and medications is essential when interpreting these results, and supplements should ideally be held prior to testing.

The optimal management of low BMD and fragility fractures in adults with HPP remains a clinical challenge, as the available evidence is limited and no guidelines currently exist to guide treatment decisions. In patients with HPP and fragility fractures and/or low bone mass, treatment should be individualized. Although some observational studies have reported the use of bisphosphonates or denosumab in this population [10], potent antiresorptives are not recommended in patients with confirmed HPP. This is due to suppression of bone turnover induced by PPi and deactivation of TNSALP. Recognition of HPP in the older adult population is critical in order to provide appropriate management and to prevent complications from treatment with antiresorptive osteoporosis medication.

In addition to non‐pharmacologic measures, anabolic osteoporosis therapy may be preferable for those with low bone mass and fragility fractures [3, 11]. Monitoring should include serial ALP measurements, symptom tracking, and imaging when indicated. A multidisciplinary team is essential to aid in management; and more studies are needed to determine optimal treatment of older patients with HPP.

Enzyme replacement therapy with asfotase alfa is available for treatment of HPP [12]. In children there is evidence for improved outcomes in gross motor function, muscle strength, and reduced functional disability [13]. Although the data is limited, benefits have been seen In studies that included adults with HPP (age range 13–78 years) [12, 13].

In patients with HPP without fractures, the clinical approach is less well defined. Some experts suggest careful monitoring without pharmacologic treatment, unless additional risk factors for fractures are present.

Differentiating osteoporosis from osteomalacia in adults with HPP can be challenging, but it is clinically relevant as the therapeutic implications differ. In HPP, osteomalacia results from defective mineralization and may present with or without low BMD [14]. However, unlike typical osteoporosis, osteomalacia may also be accompanied by stress fractures, muscle weakness, fatiguability, abnormal ALP, and radiographic signs such as Looser's zones. Clinical evaluation, laboratory testing, and imaging findings should be integrated to confirm the diagnosis. In the absence of a bone biopsy, this differentiation remains difficult and must be considered before initiating antiresorptive therapy.

In summary, HPP is an important diagnosis to consider in adults with low BMD, as there may be harms associated with using potent antiresorptive osteoporosis medications, Treatment decisions should take into account fracture risk, clinical and biochemical findings, and the possibility that low BMD may reflect osteomalacia rather than classic osteoporosis.

## Author Contributions

**Estefania Valdez Navarro:** conceptualization, investigation, project administration, software, supervision, validation, visualization, writing – original draft, writing – review and editing. **Evelyn M. Wong:** conceptualization, investigation, project administration, supervision, validation, visualization, writing – original draft, writing – review and editing. **Lianne Tile:** conceptualization, investigation, project administration, supervision, validation, visualization, writing – review and editing. **Angela M. Cheung:** funding acquisition, investigation, project administration, resources, supervision, validation, visualization, writing – review and editing.

## Ethics Statement

We confirm that this manuscript has not been published elsewhere nor is it under consideration for publication. As a case series with both patients' informed consent signed, no other ethical review was required.

## Consent

Written informed consent was obtained from both patients for the publication of this case series.

## Conflicts of Interest

A.M.C. has received honoraria as a consultant for Alexion (maker of asfotase alfa) and Amgen (maker of denosumab). All other authors have no conflicts of interest.

## Data Availability

Data sharing not applicable to this article as no datasets were generated or analyzed during the current study.
